# Bapineuzumab Alters Aβ Composition: Implications for the Amyloid Cascade Hypothesis and Anti-Amyloid Immunotherapy

**DOI:** 10.1371/journal.pone.0059735

**Published:** 2013-03-21

**Authors:** Alex E. Roher, David H. Cribbs, Ronald C. Kim, Chera L. Maarouf, Charisse M. Whiteside, Tyler A. Kokjohn, Ian D. Daugs, Elizabeth Head, Carolyn Liebsack, Geidy Serrano, Christine Belden, Marwan N. Sabbagh, Thomas G. Beach

**Affiliations:** 1 The Longtine Center for Neurodegenerative Biochemistry, Banner Sun Health Research Institute, Sun City, Arizona, United States of America; 2 Department of Neurology, University of California Irvine, Irvine, California, United States of America; 3 UCI MIND, University of California Irvine, Irvine, California, United States of America; 4 Department of Pathology, University of California Irivine, Irivine, California, United States of America; 5 Department of Microbiology, Midwestern University School of Medicine, Glendale, Arizona, United States of America; 6 Sanders-Brown Center on Aging, Department of Molecular and Biomedical Pharmacology, University of Kentucky, Lexington, Kentucky, United States of America; 7 Roberts Clinical Center, Banner Sun Health Research Institute, Sun City, Arizona, United States of America; 8 Civin Laboratory for Neuropathology, Banner Sun Health Research Institute, Sun City, Arizona, United States of America; Massachusetts General Hospital and Harvard Medical School, United States of America

## Abstract

The characteristic neuropathological changes associated with Alzheimer’s disease (AD) and other lines of evidence support the amyloid cascade hypothesis. Viewing amyloid deposits as the prime instigator of dementia has now led to clinical trials of multiple strategies to remove or prevent their formation. We performed neuropathological and biochemical assessments of 3 subjects treated with bapineuzumab infusions. Histological analyses were conducted to quantify amyloid plaque densities, Braak stages and the extent of cerebral amyloid angiopathy (CAA). Amyloid-β (Aβ) species in frontal and temporal lobe samples were quantified by ELISA. Western blots of amyloid-β precursor protein (AβPP) and its C-terminal (CT) fragments as well as tau species were performed. Bapineuzumab-treated (Bapi-AD) subjects were compared to non-immunized age-matched subjects with AD (NI-AD) and non-demented control (NDC) cases. Our study revealed that Bapi-AD subjects exhibited overall amyloid plaque densities similar to those of NI-AD cases. In addition, CAA was moderate to severe in NI-AD and Bapi-AD patients. Although histologically-demonstrable leptomeningeal, cerebrovascular and neuroparenchymal-amyloid densities all appeared unaffected by treatment, Aβ peptide profiles were significantly altered in Bapi-AD subjects. There was a trend for reduction in total Aβ_42_ levels as well as an increase in Aβ_40_ which led to a corresponding significant decrease in Aβ_42_:Aβ_40_ ratio in comparison to NI-AD subjects. There were no differences in the levels of AβPP, CT99 and CT83 or tau species between Bapi-AD and NI-AD subjects. The remarkable alteration in Aβ profiles reveals a dynamic amyloid production in which removal and depositional processes were apparently perturbed by bapineuzumab therapy. Despite the alteration in biochemical composition, all 3 immunized subjects exhibited continued cognitive decline.

## Introduction

The most common form of dementia is Alzheimer’s disease (AD), currently affecting about 24 million people worldwide with expected doubling of incidence every 20 years [1]. The disease is neuropathologically characterized by the profuse deposition of fibrillar amyloid-β (Aβ) peptides, amyloid plaques (AP) and cerebral amyloid angiopathy (CAA) as well as the intraneuronal accumulation of neurofibrillary tangles (NFT) mainly composed of tau protein. The abundance of these lesions has lent support to the amyloid cascade hypothesis as the fundamental causative incident in the pathogenesis of AD. This model has been reinforced by the fact that AP, CAA and NFT are also present in familial AD (FAD) due to presenilin (PS) and amyloid-β precursor protein (AβPP) mutations and are recapitulated in genetically-engineered transgenic (Tg) mice bearing mutated forms of AβPP, PS and tau. Furthermore, individuals with Down’s syndrome carrying 3 copies of chromosome 21, the location of the AβPP gene, also develop the neuropathology of AD.

At present, there is no effective disease-modifying treatment for AD. However, the abundant deposits of insoluble Aβ and elevated values of soluble oligomeric Aβ have prompted the design of multiple active and passive immunotherapies aimed at removing the toxic forms of these peptides [2], [3]. An example of one of the passive immunization treatments is bapineuzumab, a humanized monoclonal antibody (3D6) directed specifically against the N-terminal region of Aβ (residues 1–5) [4]. Although passive and active immunotherapies have been effective in the removal of Aβ in AβPP and PS Tg mice models, the application of these interventions to AD patients has been only partially successful from a neuropathological perspective [5]–[9], while no clear beneficial modification of the disease course has been observed in clinical trials [7], [10], [11].

In this study we assess the clinical history, neuropathological and biochemical outcomes in 3 individuals that participated in clinical trials evaluating bapineuzumab [12] (ClinicalTrials.gov Identifier NCT00112073) and who received 2, 6 or 20 immunotherapy doses. We quantified and characterized the levels of soluble and insoluble Aβ peptides that remained in the frontal and temporal lobes of bapineuzumab-immunized AD (Bapi-AD) patients and compared them to 4 age-matched non-immunized AD (NI-AD) and 4 age-matched non-demented control (NDC) individuals. AβPP and its C-terminal (CT) peptides as well as the cytokine tumor necrosis factor-α (TNF-α) were also quantified. We also compared the bapineuzumab observations with those obtained from subjects who received AN-1792 immunotherapy.

## Materials and Methods

### Clinical and Neuropathological Reports and Treatment Synopsis

The present report deals with the neuropathological and biochemical observations made on 3 Bapi-AD patients and their comparison to NI-AD and NDC individuals. Brain samples from cases #1 and #2 were obtained from the Banner Sun Health Research Institute (BSHRI) Brain and Body Donation Program [13] whose operations have been approved by the Banner Health Institutional Review Board. All subjects enrolled in the Brain and Body Donation Program sign an informed consent approved by the Banner Health Institutional Review Board. The brain tissue for case #3 was provided by the Institute for Memory Impairments and Neurological Disorders and the University of California Alzheimer’s Disease Research Center (UCI-ADRC). Participants enrolled in the UCI-ADRC provide informed consent approved by the UCI Institutional Review Board. A summary of subject demographics and neuropathology is presented in **Table 1**.

**Table 1:** Subject Demographics and Neuropathological Assessments.

NDC	Expired Age (yrs)	Gender	PMI (hrs)	Brain weight(g)	Last MMSE Score	*APOE* GT	Disease duration (yrs)	Total plaque score	Plaque density	Braak stage	CAA	Total WMR score
20	78	F	3.33	1150	28	2/3	–	4	sparse	III	Mild	2
21	90	F	4	1160	26	3/3	–	0	zero	IV	Mild	0
22	76	M	2.33	1375	29	3/4	–	5.5	sparse	I	Mild	1
23	74	M	3.25	1440	N/A	2/3	–	0	zero	I	None	2
**NI-AD**	**Expired Age (yrs)**	**Gender**	**PMI (hrs)**	**Brain weight** **(g)**	**Last MMSE Score**	***APOE*** ** GT**	**Disease duration (yrs)**	**Total plaque score**	**Plaque density**	**Braak stage**	**CAA**	**Total WMR score**
10	91	F	2	1045	N/A	3/4	7	14	frequent	V	Mild	N/A
11	84	M	2	1160	16	4/4	8	14	frequent	V	None	0
12	87	M	2.5	1100	13	2/3	6	15	frequent	VI	Severe Occ.	6
13	103	F	2.6	930	4	2/3	10	13.5	frequent	IV	Severe Occ.	7
**BSHRI Bapi-AD**	**Expired Age (yrs)**	**Gender**	**PMI (hrs)**	**Brain weight** **(g)**	**Last MMSE Score**	***APOE*** ** GT**	**Disease duration (yrs)**	**Total plaque score**	**Plaque density**	**Braak stage**	**CAA**	**Total WMR score**
1	79	F	3	1000	9	4/4	8	15	frequent	VI	Severe Occ./ pariet.	5
2	89	M	2.2	1132	21	2/3	11	12.5	frequent	V	Mod.	2
**UCI Bapi-AD**	**Expired Age (yrs)**	**Gender**	**PMI (hrs)**	**Brain weight (g)**	**Last MMSE Score**	***APOE*** ** GT**	**Disease duration (yrs)**	**Total plaque score**	**Plaque density**	**Braak stage**	**CAA**	**Total WMR score**
3	86	M	5.0	1170	0	3/4	8	N/A	frequent	VI	Mod. Occ./Frontal	N/A

The total plaque score has a maximum of 15. The total WMR score has a maximum of 12. Some neuropathological assessments are not available due to the use of different classification protocols between the 2 institutions involved in the study.

Abbreviations: NDC, non-demented control; NI-AD, non-immunized Alzheimer’s disease; BSHRI, Banner Sun Health Research Institute; Bapi-AD, bapineuzumab immunized Alzheimer’s disease; UCI, University of California, Irvine; yrs, years; F, female; M, male; PMI, postmortem interval; g, grams, MMSE, mini mental state examination; *APOE*, apolipoprotein E; GT, genotype; CAA, cerebral amyloid angiopathy; Occ., occipital; pariet., parietal; Mod., moderate; WMR, white matter rarefaction; N/A, not available.

### Case #1

For a detailed clinico-pathological description of case #1 the reader is referred to our recent publication [14]. In brief, a 79-year-old female was diagnosed with AD about 8 years prior to death. The patient received 4 doses of bapineuzumab (each dose 0.5 mg/kg) over a period of 39 weeks in the extension portion of the clinical trial. About 1 month after the last dose, the patient showed symptoms and signs of vasogenic edema on an MRI scan that was cleared prior to her death. There were no apparent signs of disease modification that could be attributed to the immunotherapy treatment. Neuropathological analysis revealed frequent AP and NFT and a concurrent diagnosis of both AD and Binswanger’s type of vascular dementia. Case #1 was incorporated in the present biochemical evaluations of cases #2 and #3 to increase the sample size. Furthermore, in the previous analysis of case #1, we only characterized the frontal region, while in this communication we added the temporal region.

### Case #2: Clinical History

An 89-year-old man died with a diagnosis of AD about 12 years after symptom onset. The available private medical records include his first presentation, about 11 years prior to death, when he was seen by a neurologist for a several month history of cognitive deterioration following biopsy for temporal arteritis. He had been getting lost even when going for a walk close to home and had been repeating himself frequently. Additionally, he was being treated for depression. On examination he was not oriented to place. He scored 23/30 on the Mini Mental State Examination (MMSE), losing points on delayed recall and orientation. Gait and posture were normal with no focal neurological signs. Brain MRI showed only ectasia of the intracranial segment of the left vertebral artery. An EEG was interpreted as within normal limits. He was started on Aricept for a presumptive diagnosis of early Alzheimer’s dementia, but this was quickly switched to Exelon because of gastrointestinal side effects. Over the next 10 years, his cognitive status declined only very slowly as documented by MMSE scores of 20/30, 19/30 and a final score of 21/30 about 1 month prior to death. Over this time period, he developed aggressive behavior and was treated with an anti-psychotic agent. He developed startle myoclonus, a stooped posture and a tremor. An EEG showed only slowing and disorganization. About 1 year prior to death, he had orthostatic hypotension with syncopal episodes and few months prior to death was hospitalized for bradycardia. He received annual standardized neurological and neuropsychological assessments at BSHRI between 2003 and 2011. The diagnostic impression was of dementia due to probable AD, with gait ataxia and essential tremor. His past medical history is otherwise notable for hyperlipidemia, mitral valve prolapse, first degree atrioventricular block, chronic obstructive pulmonary disease, glucose intolerance, thyroid nodules, bilateral cataract extractions, glaucoma and benign prostatic hypertrophy. The family history is notable for late-onset dementia in his mother.

Between January 2006 and January 2007, the patient was enrolled in the bapineuzumab clinical trial AAB-201 (Clinical Trials.Gov Identifier NCT00112073). In April 2007 the patient began to participate in the bapineuzumab open label clinical trial AAB-001, and was in this program until January 2011. During these periods, this patient received 20 infusions of bapineuzumab (each at 1 mg/kg) over a period of 260 weeks. Each infusion of bapineuzumab was administered approximately every 3 months. The apolipoprotein E (*APOE*) genotype of this individual was ε2/3.

Neuropsychological data were available for 2003, 2004, 2009, and 2011. In the memory domain, Rey AVLT total learning showed consistent decline from low average to mildly impaired. He was unable to recall any AVLT information at delay in any testing year. Recognition memory raw scores varied but were within impaired ranges across years. Narrative recall (WMS-R logical memory) was administered in 2009 and 2011 with only mild to moderate impairment noted in both years. Visual memory (BVMT-R) was administered in 2009 and 2011 only and performance was moderately to severely impaired in both years. Simple visual attention (TMTA) declined from low average to moderately impaired over four testing epochs. Simple auditory attention (Digits forward) improved from mildly impaired in 2003 and 2004 to low average in 2009 and 2011; this is a difference of one point improvement in span. Executive functions (as measured by Stroop C/W and Trail Making Test B) declined over time from low average to moderate to severe impairment. Confrontation naming reduced from 26/30 correctly identified objects in 2009 to 21/30 in 2011. Judgment of Line Orientation (JLO) was in the average range in the first 3 testing years. JLO was not administered in 2011 due to inability to complete sample items. The subject began to exhibit color discrimination problems in 2011. Consistent performance was noted on the MMSE (24, 21, 24, 21, respectively) and the clock drawing task (10/10 each year). Verbal fluency was variable across epochs. Phonemic fluency was average in 2003 and 2004, above average in 2009, and measured in the low end of the average range in 2011. Semantic fluency for animals was mildly impaired in 2003, low average in 2004, average in 2009, and low average in 2011. Over the course of 8 years, this subject generally displayed a downward trend in cognition with a few exceptions for specific tests.

### Case #2: Neuropathology Report

#### Gross examination

The brain weight at autopsy was 1132 g. The dura was normal and the leptomeninges showed moderate fibrosis. The convexities were symmetrical and showed moderate gyral atrophy of the frontal lobes, moderate to severe gyral atrophy of the parietal lobes and no gyral atrophy of the occipital lobes. No focal lesions were present on the convexities or base of the brain. The circle of Willis showed moderate patchy atherosclerosis. The mammillary bodies were normal in shape, color and size. The temporal lobes and unci showed mild to moderate gyral atrophy. The cerebellum and brainstem were externally normal. Cerebral slices showed moderate to marked enlargement of the posterior horns of the lateral ventricles and mild dilatation elsewhere. The basal ganglia, thalamus and subthalamic nucleus were unremarkable. The amygdala showed moderate atrophy in the left hemisphere and severe atrophy in the right hemisphere with compensatory enlargement of the temporal horns. The head of the hippocampus was mildly atrophied in both hemispheres. The body of the hippocampus and the parahippocampal gyrus were both mildly atrophied. The substantia nigra showed mild depigmentation bilaterally. Respective axial and parasagittal slices of the brainstem and cerebellum were normal.

#### Microscopic examination

Paraffin sections of the left hemibrain stained with hematoxylin and eosin (H&E) showed, in sections of cerebral cortex, mild to moderate upper layer gliosis. The amygdala and entorhinal cortex showed moderate to marked gliosis. Area CA1 of the hippocampus showed mild gliosis. The basal ganglia were unremarkable. There was mild to moderate gliosis of the hypothalamus. Subthalamic regions including the subthalamic nucleus and mammillary body were unremarkable. The substantia nigra showed no apparent depletion of pigmented neurons while the locus ceruleus was moderately to markedly depleted; there were no Lewy bodies present in either region. The cerebellar superior vermis showed moderate to marked patchy loss of Purkinje cells. Remaining sections of the cerebellum, brainstem and major levels of spinal cord were unremarkable. Large sections stained with H&E showed no significant cerebral white matter rarefaction and no infarcts. There were several mineralized blood vessels in the globus pallidus. Sections stained with Gallyas, Campbell-Switzer and Thioflavine-S methods showed, in neocortical regions, frequent senile plaques of the diffuse type while neuritic and cored plaques had a patchy distribution, ranging from sparse to frequent in the frontal, parietal and occipital lobes, with moderate to frequent densities in the temporal lobe. Neurofibrillary tangles were also patchily distributed, ranging from sparse to moderate to focally frequent in neocortical areas. Tangles were frequent in the amygdala, entorhinal cortex and hippocampal CA1 region. Argyrophilic grains were present at frequent densities in the amygdala, entorhinal cortex and area CA1 of the hippocampus. There were frequent Gallyas-positive glial cells around the circumference of the amygdala; these resembled small astrocytes with spiky processes. Cerebral amyloid angiopathy was present at sparse to moderate to focally frequent densities in most cerebral cortex regions while there were focally moderate densities in the cerebellar leptomeninges. Immunohistochemical staining for phosphorylated α-synuclein showed no evidence of immunoreactive inclusions or associated neurites in the olfactory bulb, brainstem, amygdala or cerebral cortex. Diagnosis: Alzheimer’s disease; argyrophilic grains and non-specific glial tauopathy, mesial temporal lobe. Comment: This microscopic examination confirms the clinical diagnosis of AD. Argyrophilic grains are a microscopic finding of uncertain significance; they occur in approximately 25% of cognitively normal older people as well as a similar fraction of those with AD and other aging brain disorders. They are often, as in this case, accompanied by a non-specific glial tauopathy.

### Case #3: Clinical History

This patient was an 86-year-old man with 12 years of education, who presented with a 4-year history of memory impairment of sudden onset prior to his initial evaluation in 1999 at the UCI-ADRC. He was followed longitudinally by the ADRC Clinical Core with slow progressive decline. The patient was screened for participation in the bapineuzumab clinical trial in August of 2006, and enrolled in this double blind randomized multicenter study with a moderate level of cognitive impairment (MMSE 17/30). This patient received only 2 doses of 2.0 mg/kg of bapineuzumab. The first infusion was given in September of 2006, followed by the second infusion 13 weeks later. MRI scans were completed for safety assessment 6 weeks after each infusion and acute vasogenic edema was observed on the safety scan at 6 weeks following the second infusion. Progressive cognitive decline occurred with a MMSE of 8/30 measured just 6 months prior to death. At autopsy, sufficient neuropathology for a diagnosis of AD with Lewy body disease was observed with a Braak stage VI. The *APOE* genotype of this individual was ε3/4.

### Case #3: Neuropathology Report

#### Gross examination

The brain weight at autopsy was 1170 g. On external examination the cerebral gyri and sulci were of normal gross appearance. The cerebral arteries and their major branches at the base of the brain showed moderate focal atherosclerosis, with up to 40% narrowing of the lumen. Coronal sections of the right cerebrum showed the presence of mild gyral narrowing and sulcal widening in the sylvian region. The lateral ventricle did not appear to be enlarged, although the hippocampus was judged to be small. Horizontal sections of the brainstem and cerebellum revealed normal intensity of neuromelanin pigmentation within the substantia nigra and markedly reduced intensity of pigmentation within the locus ceruleus.

#### Microscopic examination

Blocks of tissue were examined from middle frontal, superior temporal, inferior parietal, calcarine/pericalcarine, and rostral and caudal cingulate cortex and white matter, as well as from hippocampus, amygdala, corpus striatum, thalamus, midbrain, pons, medulla, and cerebellum. Both diffuse and neuritic (Braak and Braak stage C) plaque formation were intense within frontal, temporal, parietal, occipital, and rostral and caudal cingulate cortex and within hippocampal CA1, subiculum, entorhinal-transentorhinal region, and amygdala. Within the cerebral neocortex neuritic plaques were more prominent and densely stained (in both modified Bielschowsky and Aβ-stained sections) within the superficial layer than within deeper layers while the number of plaques appeared to be similar, but lighter in stain intensity. These findings were also observed within the hippocampal formation, particularly within the subiculum and, even more strikingly, within the amygdala, where the intensity of Aβ immunostaining within many of the neuritic plaques was markedly reduced. Vascular intramural Aβ deposition was also observed in moderate degree within frontal and occipital leptomeningeal and intracortical vessels and, in very mild degree, within amygdala capillaries. Iron deposition, as seen with the aid of Perl’s iron stains, was minimal. Neurofibrillary degeneration, which was judged overall Braak stage VI was severe at all sites examined except for the calcarine cortex, where it was absent. Granulovacuolar degeneration and Hirano body formation were readily observed within hippocampal CA1. The concentration of neuromelanin-bearing neurons was normal within substantia nigra and moderately severely reduced within locus ceruleus. Alpha-synuclein-immunoreactive intracytoplasmic Lewy inclusion bodies were observed within substantia nigra, amygdala, subiculum, entorhinal cortex and rostral cingulate cortex.

### Neuropathological Evaluation

Bapi-AD cases #1 and #2, NI-AD and NDC subjects (BSHRI) were evaluated for total plaque score, amyloid plaque density, total CAA score, total NFT score, white matter rarefaction (WMR) score, CERAD criteria, neuritic plaque score [15] and Braak stage [16] as described in detail in previous publications [13], [17], [18]. As part of the standard UCI-ADRC Neuropathology Core protocol, blocks of fixed tissue from Bapi-AD case #3 were embedded in paraffin and sectioned at 8 µm for a final standard neuropathological diagnosis (NIA Reagan criteria [19]). Modified Bielschowsky, H&E, and Klűver-Barrera stains and immunostains for tau, α-synuclein, ubiquitin, glial fibrillary acidic protein (GFAP - astrocytes) and CD68 (microglia) were applied using standard immunohistochemical protocols [20], [21]. Braak staging was assessed using silver-stained sections [16]. For additional experimental studies, tissue blocks containing middle frontal gyrus and hippocampus were sectioned with a vibratome at 50 µm intervals. Standard immunohistochemical methods were used to detect Aβ_1–40_ and Aβ_1–42_ (Biosource Internationial, Camarillo, CA) after pretreatment with 90% formic acid for 4 min [22]. Thioflavine-S (0.1%) staining was used to visualize whether existing plaques containing Aβ were fibrillar.

### Assessment of Cerebrovascular Amyloid Angiopathy (CAA)

To investigate the degree of vascular amyloidosis in the leptomeninges, these membranes were carefully removed from the convexities and medial aspect of the cerebral hemispheres, prior to the brain coronal sectioning. The leptomeninges were rinsed in phosphate buffered saline (PBS) and frozen at −80°C until the moment of utilization. To remove entrapped intravascular blood, the leptomeninges were rinsed 6 times each with 2 L of cold PBS, and a final rinse with 2 L of deionized water (DW). The gleaming membranes were spread on the surface of 3 plastic Petri dishes (14 cm diameter) and allowed to dry and adhere to the plastic surface by heating in an oven at 65°C. The membranes were fixed with absolute ethanol for 1 h, rinsed with DW and stained with 1% aqueous Thioflavine-S for 15 min followed by 4 rinses with 70% ethanol to remove unbound fluorochrome and immediately observed in an epifluorescence microscope. For the appraisal of cortical vascular amyloid, 0.5 cm^3^ of cerebral cortex were suspended in 600 ml of 50 mM Tris-HCl pH 7.5 buffer containing 5% SDS and 0.01% sodium azide with continuous stirring for 72 h until all tissue was lysed except the tufts of insoluble blood vessels. The blood vessels were rinsed with 4 L of DW, to remove excess SDS, and processed and stained as described above for the leptomeninges.

### Immunohistochemistry

Coronal free-floating, formalin-fixed sections of 40 µm thickness were taken using a sliding freezing microtome from Bapi-AD cases #1 and #2, NI-AD and NDC subjects and were washed in PBS, 0.3% Triton X-100 (PBS-Tx) buffer to remove the cryoprotectant storage medium. For diaminobenzidine (DAB) signal development, the sections were blocked for 30 min in 1% H_2_O_2_, washed with PBS-Tx and incubated for 16 h at room temperature in their respective primary antibodies supplied by Abcam: anti-CD68 (ab955; 1∶1000 dilution), anti-anti-HLA-DR (ab2018; 1∶1000 dilution) and anti-CD3 (ab16669; 1∶500 dilution). Sections were then washed in PBS-Tx, incubated in the appropriate secondary biotinylated antibody for 2 h, washed with PBS-Tx and incubated in avidin-biotin peroxidase (ABC,Vector Labs) for 30 min. The sections were placed in DAB for 3–8 min transferred to Tris-buffer and mounted onto glass slides followed by a counterstain in 1% neutral red and dehydration. The sections were coverslipped with Permount mounting media (Fisher Scientific, Pittsburg, PA). For fluorescence staining, the sections were reacted with rabbit anti-GFAP (ab7260: Abcam, Cambridge, MA) at a 1∶3000 dilution, incubated at room temperature for 20 h on an orbital shaker and then washed 6 times with PBS-Tx. The sections were incubated with Alexa Fluor 488-conjugated goat anti-rabbit IgG for 2 h (A-11034: Life Technology Corp. Carlsbad CA) at a 1∶1000 dilution followed by 6 washes in PBS-Tx and the sections mounted on glass slides and dried. The slides were then sequentially submersed in 70% ethanol, 1% Sudan black in 70% ethanol, 50% ethanol, rinsed with dH_2_O and mounted with Vectashield hard mount media (Vector Labs, Burlingame CA).

### Quantification of Soluble and Insoluble Aβ by ELISA

A detailed description of ELISA methods has been previously published [23]. In brief, gray matter from the frontal and temporal lobes (100 mg) was gently homogenized in 800 µl of 20 mM Tris-HCl, 5 mM EDTA, pH 7.8, protease inhibitor cocktail (PIC, Roche Diagnostics, Mannheim, Germany), centrifuged at 435,000×*g* and the Tris-HCl-soluble supernatant collected. The pellet was reconstituted in 600 µl of 90% glass-distilled formic acid (GDFA), centrifuged at 435,000×*g* and the supernatant collected, dialyzed against deionized water followed by 0.1 M ammonium bicarbonate to remove the GDFA, then lyophilized. The lyophilized material was reconstituted in 500 µl 5 M guanidine hydrochloride (GHCl), 50 mM Tris-HCl, pH 8.0, PIC (Roche), centrifuged and the supernatant collected. Total protein was determined for the Tris- and GDFA/GHCl-soluble extracts with Pierce’s Micro BCA protein assay kit. Aβ_40_ and Aβ_42_ were quantified with ELISA kits from Invitrogen (Carlsbad, CA) according to manufacturer instructions.

### Quantification of Tumor Necrosis Factor-α (TNF-α) by ELISA

Gray matter (100 mg) was homogenized in 10 volumes of 20 mM HEPES, 1.5 mM EDTA, pH 7.4, PIC (Roche) as previously communicated [23] and total protein concentrations determined (BCA protein assay, Pierce). Human TNF-α levels were quantified using an ELISA kit (PromoKine, Heidelberg, Germany) following the manufacturer’s directions.

### Western Blot Analysis

The complete materials and methods for Western blots have been published elsewhere [23]. The antibodies applied for these experiments were CT20AβPP (against the last 20 amino acids of AβPP; #SIG-39152, Covance, Princeton, NJ) and tau (HT7 clone against amino acids 159–163 of tau; #MN1000, Pierce, Rockford, IL). For the secondary antibody, either HRP conjugated AffiniPure goat-anti mouse IgG (#111-035-144, Jackson ImmunoResearch, West Grove, PA) or HRP conjugated AffiniPure goat-anti rabbit IgG, (#111-035-146, Jackson ImmunoResearch) was used. In addition, α-mouse actin (#A65020, BD Transduction Laboratories, San Jose, CA) or α-rabbit actin (#Ab37063, Abcam, Cambridge, MA) was used as a total protein loading control.

### Statistical Analyses

Statistical calculations were performed with GraphPad Prism 5 software (La Jolla, CA) using a one-way ANOVA followed by Tukey’s multiple comparison test. Statistical significance was determined by *p* values of ≤0.05.

## Results

**Figure 1:**
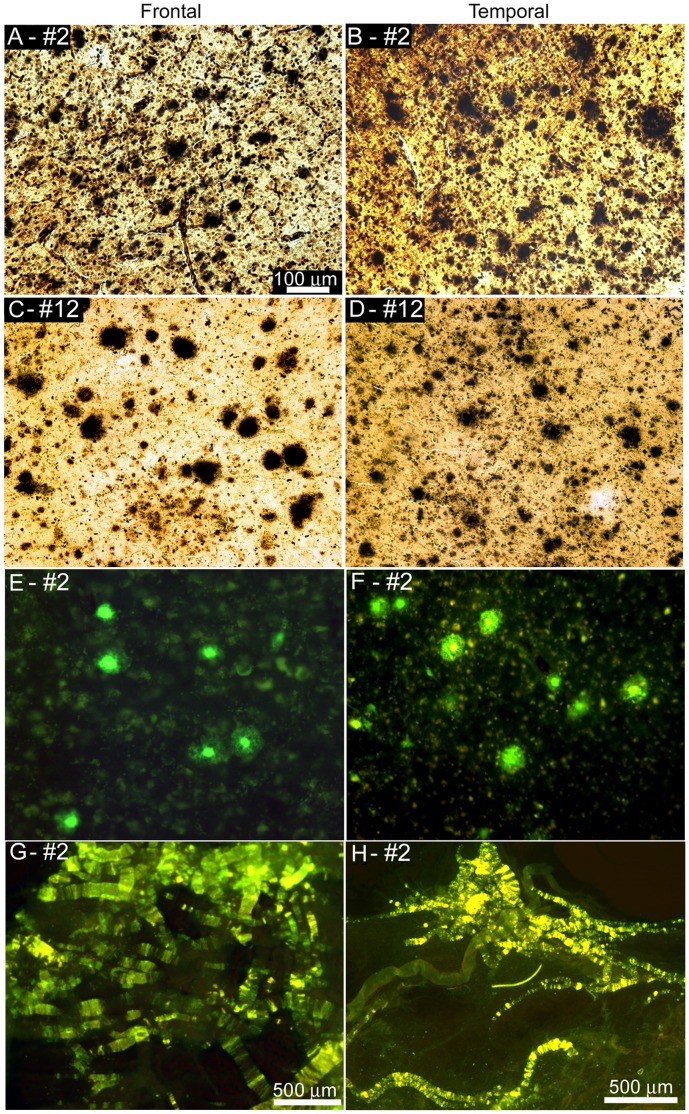
Amyloid plaques and vascular amyloid in Bapi-AD and NI-AD. Amyloid plaques in frontal (**A**, **C** and **E**) and temporal (**B**, **D** and **F**) lobes and vascular amyloid (**G** and **H**). **A**) and **B**) Campell-Switzer staining of Bapi-AD case #2 (89 year old male, *APOE* ε2/3 genotype). **C)** and **D)** Campbell-Switzer staining of NI-AD case #12 (87 year old male, *APOE* ε2/3). **E**) and **F**) Thioflavine-S staining of Bapi-AD case #2. Frequent amyloid plaques are shown in all cases. **G**) Leptomeninges of Bapi-AD case #2 stained with Thioflavine-S. **H**) Cortical blood vessels of Bapi-AD case #2 stained with Thioflavine-S. **A–F** were taken at 100× magnification and the scale bar is shown in caption **A**. **G** and **H** were taken at 25X with the scale bars shown in each caption.

**Figure 2:**
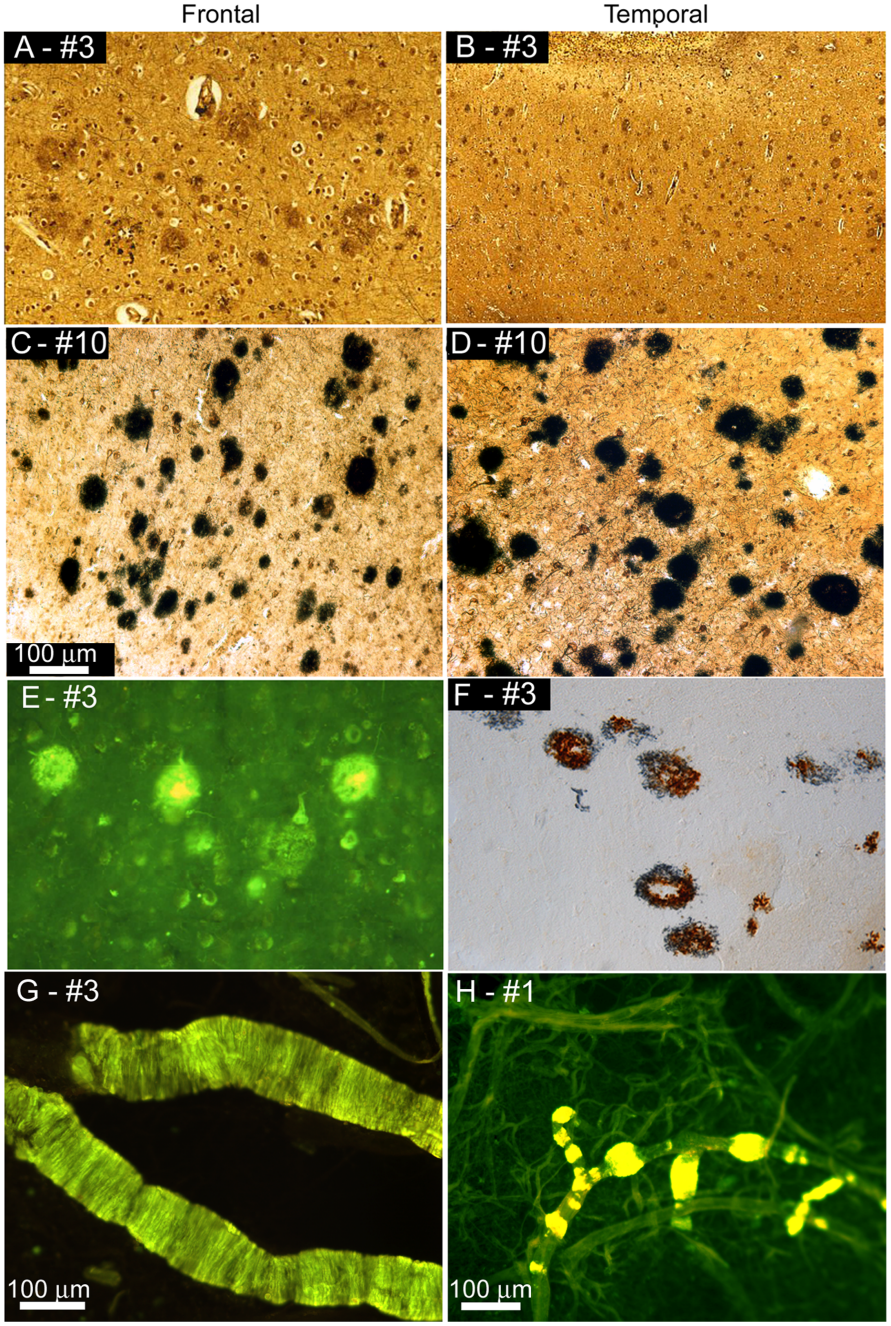
Amyloid plaques and vascular amyloid in Bapi-AD and NI-AD. Amyloid plaques in frontal (**A, C, E**) and temporal (**B, D, F**) lobes and vascular amyloid (**G** and **H**). **A**) and **B**) Bielschowsky stain of Bapi-AD case #3 (86 year old male, *APOE* ε3/4 genotype), showing a moderate amyloid plaque accumulation. **C**) and **D**) Campbell-Switzer staining of NI-AD case #10 (91 year old female, *APOE* ε3/4 genotype), exhibiting frequent amyloid plaques. **E**) Thioflavine-S staining in the frontal cortex of case of Bapi-AD case #3 showing amyloid plaques and NFT in the background. **F**) Double immunolabeling of amyloid plaques of Bapi-AD case #3 with antibodies against Aβ_40_ (brown) and Aβ_42_ (blue) demonstrating that the amyloid plaques contained both peptide species. Cortical blood vessels of Bapi-AD case #3 (**G**) and Bapi-AD case #1 (**H**) stained with Thioflavine-S. Magnifications: **A, C, D, E, F** –100X and **B** –40x.

**Table** **1** shows a detailed account of the ages, gender, postmortem intervals, brain weights, last MMSE scores, *APOE* genotypes and neuropathological assessments of the NDC, NI-AD and Bapi-AD cases employed in this study. In each of the NDC and NI-AD groups there were 2 females and 2 males, while in the Bapi-AD group there was 1 female (case #1) and 2 males (cases #2 and #3). The mean ages of the individuals in these groups were 80, 91, and 85 years, respectively. The average brain weight of the Bapi-AD individuals was 1101 g which was very close to the average weight of the NI-AD cases (1059 g), suggestive of some degree of atrophy when compared to the mean weight of the NDC group (1281 g). The Bapi-AD subjects had a final MMSE mean value of 10 while the NI-AD and NDC MMSE values were 11 and 28, respectively. The *APOE* ε4 allelic frequencies in the 3 groups under study were: NDC = 13%, NI-AD = 38% and Bapi-AD = 50%, respectively. The AP densities were zero to sparse in the NDC cases while they were frequent in the NI-AD and Bapi-AD cases **(** **Table 1** **)**, as can be appreciated in **Figure 1** and **Figure 2**. Amyloid plaques were elevated between NDC and NI-AD/Bapi-AD groups (**Table 1**). Neurofibrillary tangle Braak stages were similar between NI-AD and Bapi-AD individuals (**Table 1**). There was a wide variation in CAA content **(** **Table 1** **)**: none to mild in NDC, none to mild in NI-AD (with severe in the occipital lobe of cases #12 and #13) and moderate to severe in the case of the Bapi-AD (severe in the occipital and parietal lobes of case #1). As shown in **Table 1**, there was a relatively wide variation in total WMR score among the 3 groups.

As can be appreciated from **Figure 1A, B, E, F** and **Figure 2A, B, E, F**, based on semi-quantitative visual analyses, the Bapi-AD subjects harbored AP densities similar to those observed in NI-AD cases **(** **Figure 1C and D** **and** **Figure 2C and D** **)**, which were matched for *APOE* genotype. No histological evidence of extensive AP removal, patchy or otherwise, was visually apparent in the 3 Bapi-AD subjects. Furthermore, the density of CAA in the isolated vascular network of cerebral cortices, stained by Thioflavine-S, demonstrated moderate to severe deposition of amyloid in all 3 cases (**Figures 1H** **,** **2G and 2H**). Whole-mount histological preparations of the entire leptomeningeal vascular arbors of case #2, stained by Thioflavine-S, showed moderate to severe presence of vascular amyloid distributed in a very homogeneous fashion (**Figure 1G**). Our previously published report on Bapi-AD [14], which corresponds to case #1 of the present study, showed severe leptomeningeal CAA (see Figure 1D in reference [14]). Leptomeningeal Aβ was not assessed for case #3.

**Figure 3:**
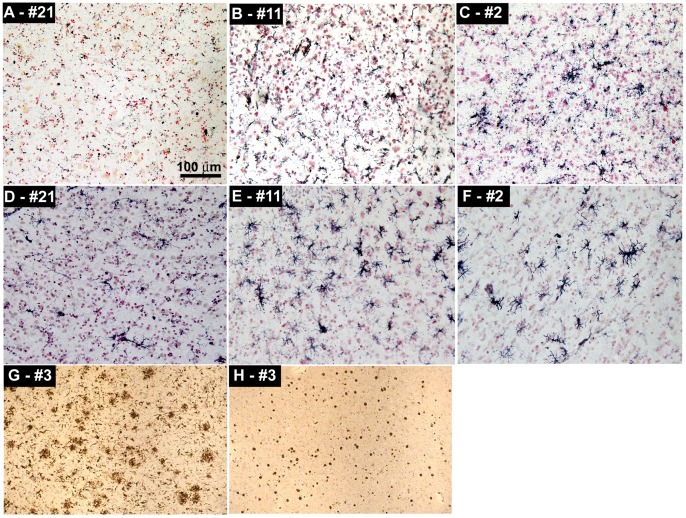
Representative images of immunohistochemistry showing microglia and T-lymphocytes. **A)** CD68 staining of the microglia in the temporal cortex of NDC case #21. **B)** CD68 staining of the temporal cortex of NI-AD case #11. **C)** CD68 staining of the temporal cortex of Bapi-AD case #2. **D)** HLA-DR staining of microglia in the frontal cortex of NDC case #21. **E)** HLA-DR staining of the temporal cortex of NI-AD case #11. **F)** HLA-DR staining of the temporal cortex of Bapi-AD case #2. **G)** HLA-DR staining of the temporal cortex of Bapi-AD case #3. **H)** CD3 staining of T-lymphocytes in the temporal cortex of Bapi-AD case #3. Magnifications: **A–F** –200X and **G**, **H** –100X.

**Figure 4:**
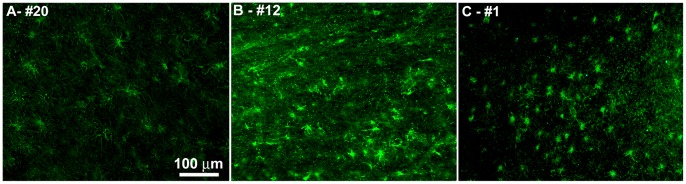
Representative images of immunofluoresence staining of GFAP. **A)** Frontal cortex of NDC case #20. **B)** Temporal cortex of NI-AD case #12. **C)** Temporal cortex of Bapi-AD case #1. For more details, see the Results section. Magnifications: **A–C** –200X.

The Bapi-AD cases #1 and #2 exhibited a dense distribution of microglia in the frontal and temporal cortices, as stained by the CD68 antibody, relative to the NDC cases; however the microglial density did not differ between Bapi-AD cases and NI-AD cases **(** **Figures 3A, B and C** **)**. This observation was confirmed by the HLA-DR antibody **(** **Figure 3D, E and F** **)**. HLA-DR immunoreactive activated microglia were seen in abundance within cerebral cortex and white matter of Bapi-AD case #3, and were even more numerous within subiculum and, particularly, within the amygdala **(** **Figure 3G** **)**. Cases #1 and #2 showed no T-cell lymphocyte infiltration in either the frontal and temporal cortices. In contrast, the Bapi-AD case #3 showed sparse but unequivocal infiltration by CD3-immunoreactive T-lymphocytes, typically in perivascular location, within cerebral cortex and white matter. T-lymphocytes were observed in higher concentrations within subiculum and, even more strikingly, within the amygdala **(** **Figure 3H** **)**. Immunofluorescence staining with GFAP demonstrated fibrous astrocytes **(** **Figure 4A** **)**. The NI-AD cases **(** **Figure 4B** **)** appeared to have more voluminous astrocytic cytoplasm and more frequent processes than the Bapi-AD group **(** **Figure 4C** **)**. Despite the higher levels of the proinflammatory cytokine TNF-α in the temporal lobe of the Bapi-AD cases relative to the NI-AD cohort, we did not observe commensurate changes in the temporal lobe microglia morphology or density.

**Figure 5:**
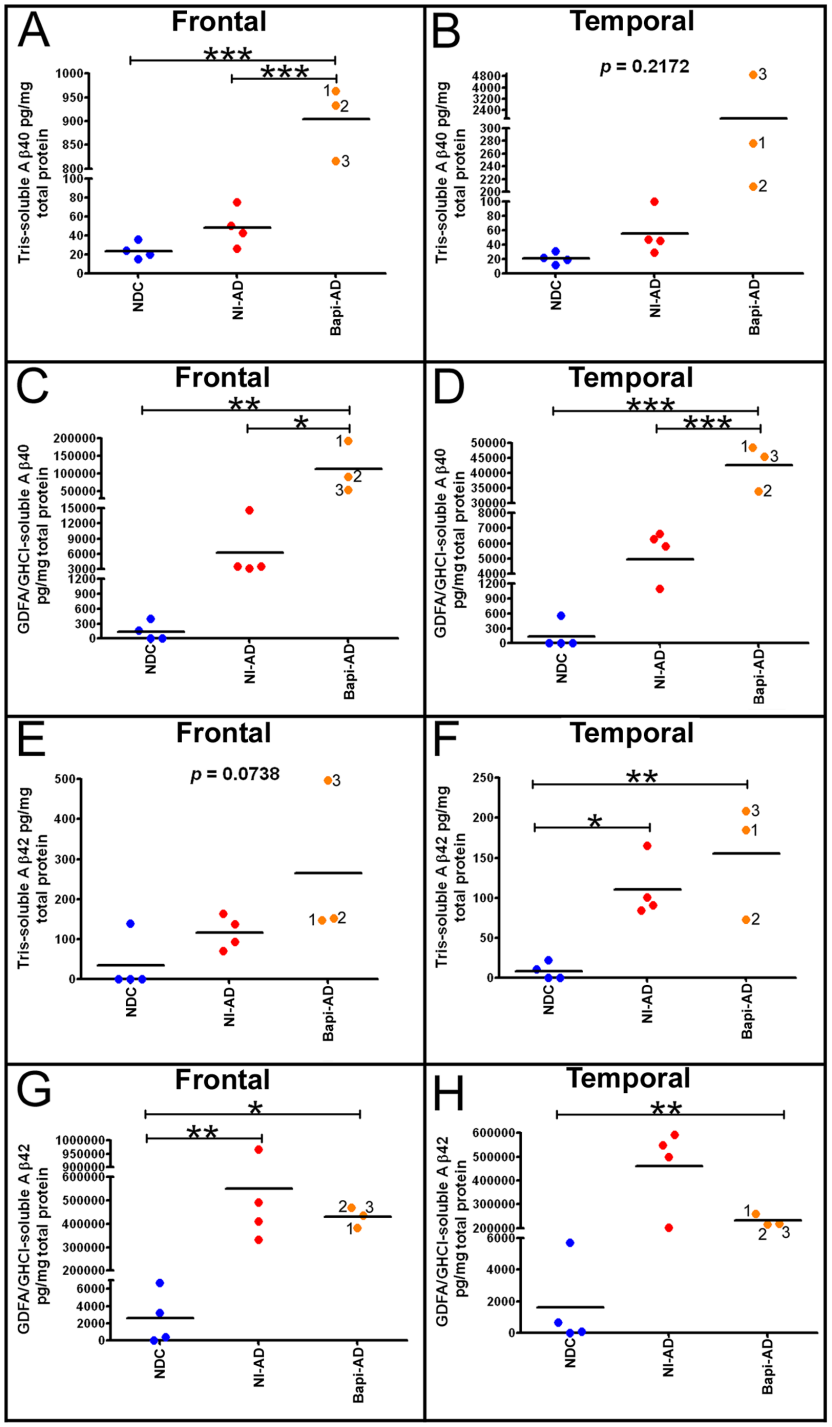
ELISA quantification of soluble and insoluble Aβ in frontal and temporal lobes. **A)** Frontal cortex Tris-soluble Aβ_40_. Notice the sharp and significance difference between the Aβ levels of NI-AD and those of the Bapi-AD. **B)** Temporal cortex Tris-soluble Aβ_40_. There was a large mean difference between NI-AD and Bapi-AD, however there was not a statistically significant difference due to the spread of values. Frontal (**C**) and temporal (**D**) cortices GDFA/GHCl-soluble Aβ_40_. In both cases there were significant elevations in mean Aβ_40_ in Bapi-AD relative to NI-AD. Frontal (**E**) and temporal (**F**) cortices Tris-soluble Aβ_42_. In both lobes there were no statistical differences between the NI-AD and Bapi-AD due to the spread in values. Frontal (**G**) and temporal (**H**) cortices GDFA/GHCl-soluble Aβ_42_. There was a mean decrease in Bapi-AD Aβ_42_ relative to NI-AD more noticeable in the latter than in the former. The numbers in the Bapi-AD columns (1, 2 and 3), correspond to the cases #s given in **Table 1**. Statistical analysis was performed using a one-way ANOVA followed by Tukey’s multiple comparison test (*p = 0.05–0.01; **p = 0.01–0.001; ***p<0.0001). Abbreviations: Tris, 20 mM Tris-HCl, 5 mM EDTA, pH 7.8, plus protease inhibitor cocktail; GDFA, glass-distilled formic acid; GHCl, 5 M guanidine hydrochloride, 50 mM Tris-HCl, pH 8.0, plus protease inhibitor cocktail; NDC, non-demented control; NI-AD, non-immunized Alzheimer’s disease; Bapi-AD, bapineuzumab-immunized Alzheimer’s disease.

Quantification of Aβ_40_ peptides by ELISA revealed that in the frontal lobe and temporal lobe **(** **Figures 5A and 5B** **)** the Tris-soluble fraction was, on the average, strongly increased in the Bapi-AD cases relative to NI-AD by 18-fold and 32-fold, respectively, although only the frontal lobe levels **(** **Figure 5A** **)** reached statistical significance (*p*<0.05). In the temporal lobe, the soluble Aβ_40_ was not statistically significant due to the spread of the values in the Bapi-AD **(** **Figure 5B** **)**. In comparison, the NI-AD group had only 2.0-fold (frontal lobe) and 2.7-fold (temporal lobe) increases in amounts of Tris-soluble Aβ_40_ as compared to the NDC cohort **(** **Figures 5A and 5B** **)**. A similar, but not as dramatic, situation was observed for the Tris-extracted Aβ_42_ pool where the average levels were 2.3- fold and 1.4-fold increased in the frontal and temporal lobes respectively, between the NI-AD and Bapi-AD cases **(** **Figure 5E and 5F** **)**.

Comparisons between Bapi-AD and NI-AD groups revealed an apparently selective removal of GDFA/GHCl-solubilized fibrillar Aβ_42_, **(** **Figure 5G and 5H** **)**, coupled with a concomitant increase of insoluble fibrillar Aβ_40_ **(** **Figure 5C and 5D** **)**. In the frontal lobe, the mean GDFA/GHCl-soluble Aβ_42_ level in the Bapi-AD group was 429 ng/mg total protein, while the mean level in the NI-AD group as compared to 549 ng/mg total protein in the NI-AD group, a 1.3-fold decrease in immunized cases **(** **Figure 5G** **)**. In contrast, the mean GDFA/GHCl-soluble Aβ_40_ levels were elevated 18-fold in the Bapi-AD group compared to the NI-AD subjects (113 ng/mg and 6.2 ng/mg, respectively) **(** **Figure 5C** **)**. In the temporal lobe, a more dramatic apparent relative loss of GDFA/GHCl-soluble Aβ_42_ was evident, decreasing from 461 ng/mg in the NI-AD cases to an average of 231 ng/mg in the Bapi-AD cases, a 2-fold reduction **(** **Figure 5H** **)**. Mean GDFA/GHCl-soluble temporal Aβ_40_ levels increased from 5.0 ng/mg in the NI-AD group to a mean of 43 ng/mg in the Bapi-AD cases, an 8.6-fold increase **(** **Figure 5D** **)**.

**Figure 6:**
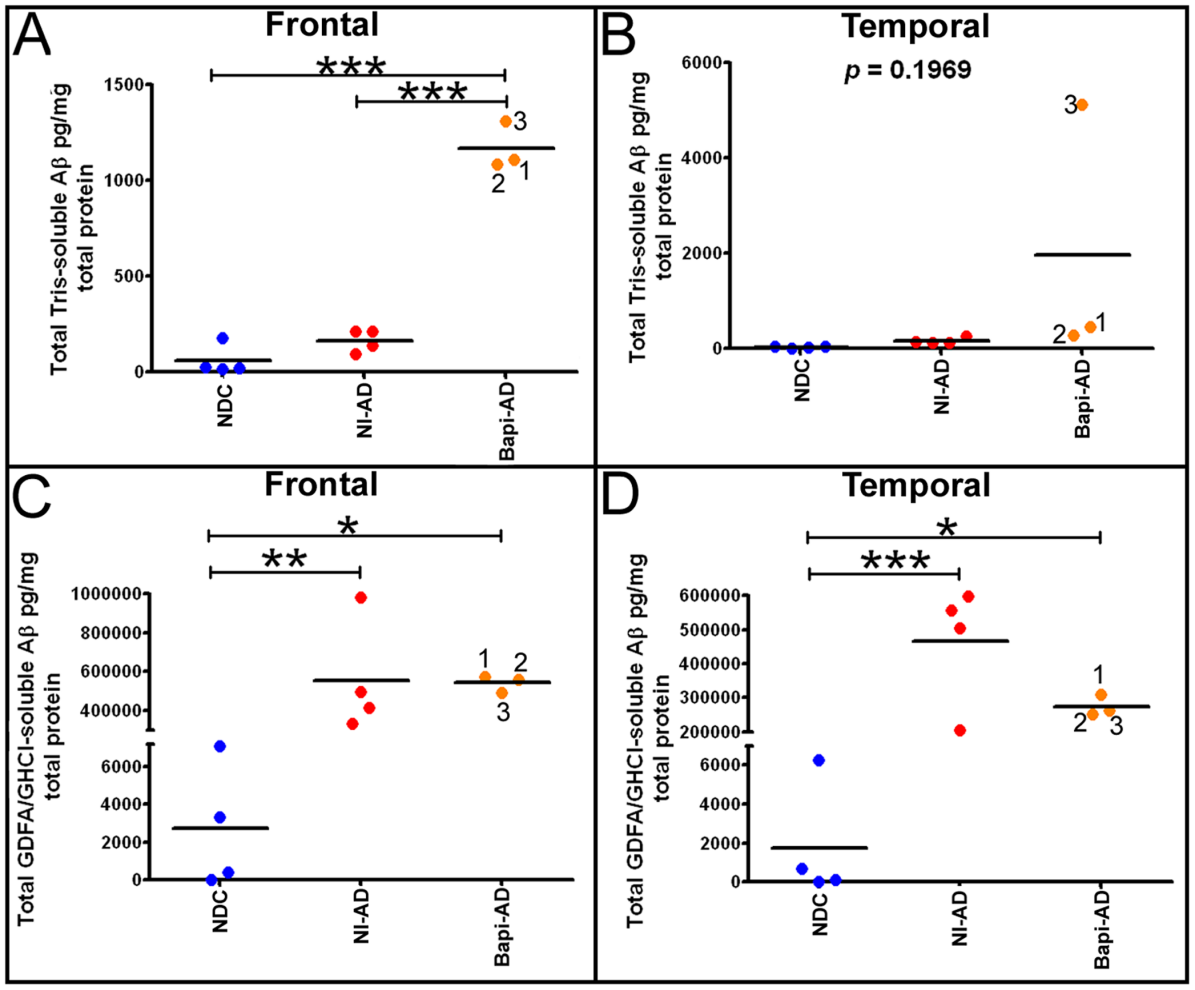
Total levels (Aβ_40_+Aβ_42_) of soluble (Tris) and insoluble (GDFA/GHCl-soluble) Aβ from the frontal and temporal lobes. **A**) Frontal cortex total Tris-soluble Aβ. **B)** Temporal cortex total Tris-soluble Aβ. **C**) Frontal cortex total GDFA/GHCl-soluble Aβ. **D**) Temporal cortex total GDFA/GHCl-soluble Aβ. For statistical treatment and abbreviations see legend to Figure 5.

Remarkably, Aβ_40_ levels increased while Aβ_42_ was decreased in the Bapi-AD subjects relative to the NI-AD cohort, resulting in no significant net change of GDFA/GHCl-soluble total Aβ levels in the Bapi-AD compared to NI-AD subjects **(** **Figure 6C and 6D** **)**. On the other hand, the levels of Tris-soluble total Aβ were significantly increased in the frontal cortex of the Bapi-AD cohort compared to NI-AD **(** **Figure 6A** **)**. Only Bapi-AD case #3 had elevated Tris-soluble total Aβ relative to the other 2 Bapi-AD subjects in the temporal lobe **(** **Figure 6B** **)**. This profound shift in Aβ_40_ accumulation in the Bapi-AD cohort was also evident in the GDFA/GHCl-soluble fractions from the frontal and temporal lobes in which Aβ_42_:Aβ_40_ ratios shifted dramatically after immunotherapy.

For the frontal and temporal lobes, the average Aβ_42_:Aβ_40_ ratios in the GDFA/GHCl-soluble fraction were 107 and 112 in the NI-AD group, respectively. These ratios were significantly decreased to 5.0 and 5.5 in the Bapi-AD cases, respectively **(** **Table 2** **)**. Similarly, as shown in **Table 2**, the Tris-soluble average Aβ_42_:Aβ_40_ ratios were significantly reduced in the immunized cases relative to the NI-AD cases (Bapi-AD = 0.31 vs. NI-AD = 2.51; 8.1-fold) in the frontal lobe, and in the temporal lobe (Bapi-AD = 0.35 vs. NI-AD = 2.19; 6.3-fold).

**Table 2:** Ratio of Aβ_42_:Aβ_40_, as measured by ELISA, in the frontal in temporal lobes of NI-AD and Bapi-AD subjects.

FRONTAL			TEMPORAL		
NI-AD	Ratio Tris-soluble Aβ_42_:Aβ_40_	Ratio GDFA/GHCl-soluble Aβ_42_:Aβ_40_	NI-AD	Ratio Tris-soluble Aβ_42_:Aβ_40_	Ratio GDFA/GHCl-soluble Aβ_42_:Aβ_40_
10	2.73	131	10	3.14	185
11	1.84	66	11	1.83	87
12	2.19	93	12	1.65	101
13	3.28	137	13	2.15	75
**Mean**	**2.51**	**107**	**Mean**	**2.19**	**112**
**Bapi-AD**	**Ratio Tris-soluble Aβ_42_:Aβ_40_**	**Ratio GDFA/GHCl-** **soluble Aβ_42_:Aβ_40_**	**Bapi-AD**	**Ratio Tris-soluble Aβ_42_:Aβ_40_**	**Ratio GDFA/GHCl-soluble Aβ_42_:Aβ_40_**
1	0.154	2.00	1	0.67	5.36
2	0.163	5.11	2	0.35	6.39
3	0.608	7.93	3	0.04	4.78
**Mean**	**0.308**	**5.01**	**Mean**	**0.35**	**5.51**
* ***p*** ** = **	**0.0025**	**0.0037**		**0.0073**	**0.0153**

Abbreviations: NI-AD, non-immunized Alzheimer’s disease; Bapi-AD, bapineuzumab-immunized Alzheimer’s disease; GDFA/GHCl, glass-distilled formic acid/5 M guanidine-hydrochloride buffer.

* Unpaired 2-tailed, t-test.

**Figure 7:**
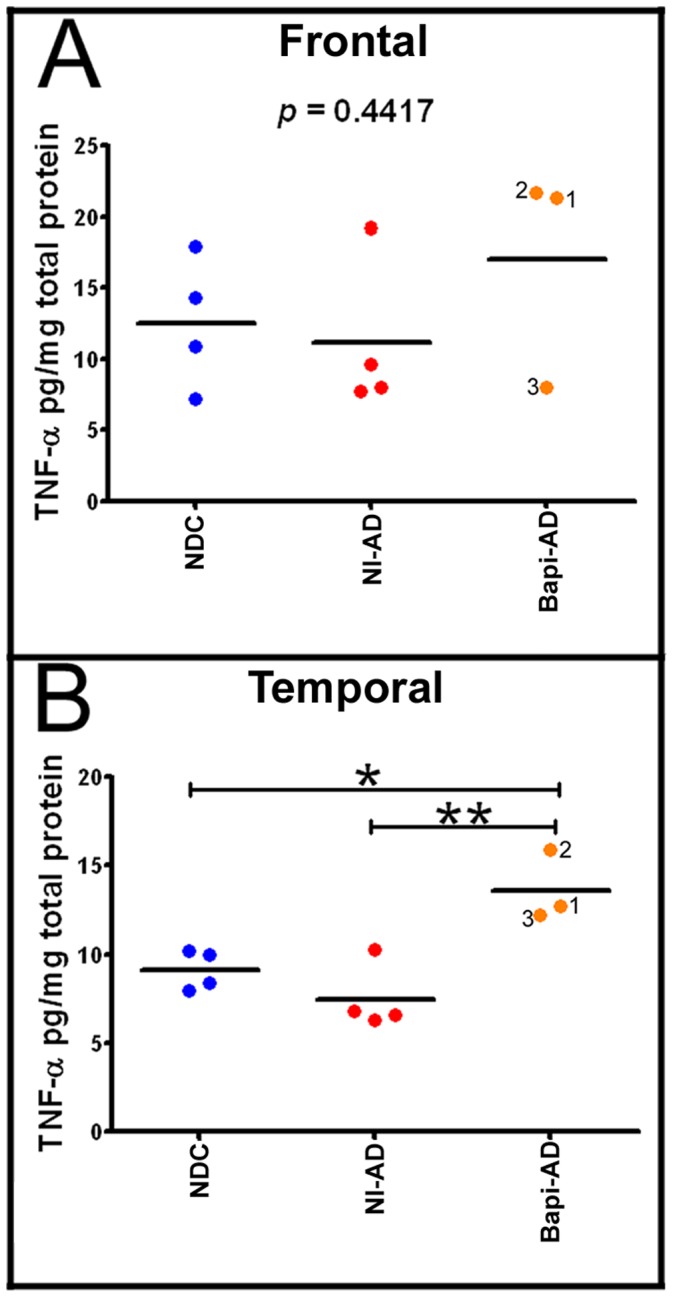
ELISA quantification of tumor necrosis factor-α (TNF-α). **A**) Frontal cortex TNF-α levels and **B**) Temporal cortex TNF-α levels. Notice that there is a significant increase in the amount of this cytokine in the temporal lobe of the Bapi-AD relative to NI-AD. For statistical treatment and abbreviations see legend to Figure 5.

In the frontal and temporal lobes the pro-inflammatory TNF-α molecule was on the average increased in the Bapi-AD cases relative to the NI-AD cases, but this difference reached a level of statistical significance only in the temporal lobe **(** **Figures 7A and 7B** **)**. As described above, removal of Aβ in the Bapi-AD was more extensive in the temporal lobe in comparison to the NI-AD group which could explain the relative increase in this pro-inflammatory cytokine.

**Figure 8:**
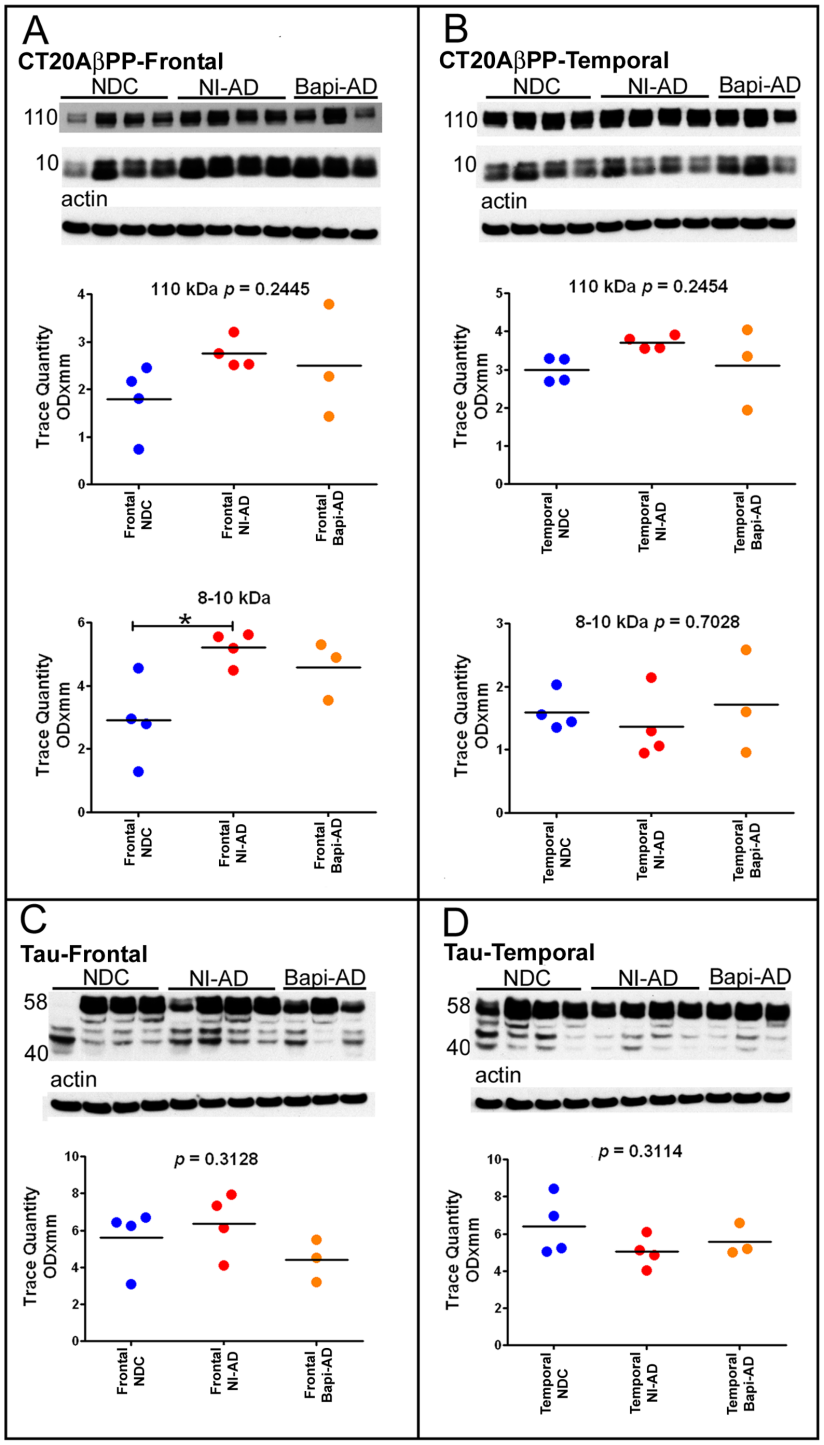
Western blot analyses of AβPP and its C-terminal peptides CT99, CT83 and of the tau isoforms. Frontal (**A**) and temporal (**B**) cortices Western blots of CT20AβPP. The CT20AβPP antibody, raised against the last 20 amino acids of AβPP, was used to detect AβPP and its CT peptides. Frontal (**C**) and temporal (**D**) cortices Western blots of tau. The tau isoforms were probed using the HT7 clone made against amino acids 159–163 of the tau molecule. Molecular weights, in kDa, are given on the left of each blot. Actin re-probes are shown below as a total protein loading control. For comparison all Western blots were analyzed against NDC and NI-AD in the same gel. There were no statistical differences between the immunized and non-immunized groups. Statistical analysis and abbreviations are described in the legend to Figure 5.

Evaluation of the AβPP content by Western blot using the CT20AβPP antibody revealed no statistically significant differences in the frontal and temporal lobes between the Bapi-AD and NI-AD cases **(** **Figures 8A and 8B** **)**. The CT20AβPP antibody also detects the CT99 (∼11 kDa) and CT83 (∼9 kDa) **(** **Figures 8A and 8B** **)**, the peptides derived from the AβPP proteolysis by the β- and α-secretases, respectively. These peptides were significantly increased in the NI-AD frontal lobe when compared to the NDC **(** **Figure 8A** **)**, but showed no differences from the Bapi-AD group. Identification of total tau by the HT7 antibody revealed the expected multiplicity of isoforms ranging from ∼58 to ∼40 kDa. However, the overall quantities of these isoforms did not show statistical differences among the studied groups or brain regions **(** **Figures 8C and 8D** **)**.

## Discussion

Our investigation revealed: **1)** On the basis of histology and number, APs were apparently undisturbed in the Bapi-AD individuals compared to age- and *APOE* genotype-matched NI-AD cases; **2)** Microgliosis and astrogliosis in the Bapi-AD and NI-AD cases were increased relative to the NDC cohort while T-cell lymphocyte infiltration was only present in Bapi-AD case #3; **3)** In the Bapi-AD subjects, the total amounts of Aβ_42_ were reduced relative to the Aβ_40_ levels, which were concomitantly elevated; **4)** A remarkable reduction in the Aβ_42_:Aβ_40_ ratios between the Bapi-AD and NI-AD cohorts; and **5)** A lack of noticeable difference in the relative levels of AβPP, CT99 and CT83 between Bapi-AD and NI-AD despite the alterations in Aβ_42_:Aβ_40_ ratios. It is unclear whether the shift in Aβ peptide composition following immunotherapy represents a true compensatory physiological response to AP and CAA disaggregation or simply reflects a new equilibrium due to continuous AβPP processing under changed environmental circumstances. Interestingly, in the Bapi-AD patients there was an apparent increase in the Tris-soluble Aβ pool relative to those levels observed in the NI-AD cases. The pathophysiological consequences of increased soluble Aβ levels that are unable to exit the brain are unknown.

A detailed examination of the individual responses to bapineuzumab in terms of the amount of antibody per infusion and number of administered doses relative to Aβ peptide levels did not show a clear pattern for the Tris-soluble Aβ fractions in both the frontal and temporal lobes. However, in the Bapi-AD individuals the levels of the GDFA/GHCl-soluble Aβ_40_ and Aβ_42_ fractions had the tendency to cluster together.

Germane to the present study are our previous observations of similar alterations in the Aβ_42_:Aβ_40_ ratio in the gray matter of the frontal lobes of 7 patients actively immunized with the AN-1792 (containing the Aβ_42_ peptide) vaccine which were compared to 6 NI-AD subjects [5]. ELISA of the GDFA/GHCl-extracted Aβ also revealed an average relative loss of Aβ_42_ and a concomitant average increase in Aβ_40_ in the AN-1792 subjects. The average Aβ_42_:Aβ_40_ ratios were 17.8 for the NI-AD and 0.59 for the AN-1792 subjects, thus demonstrating trends analogous to those exhibited in the current bapineuzumab study. As a group, the AN-1792-immunized subjects demonstrated an average GDFA/GHCl-soluble total Aβ loss of 9.3%. Furthermore, the total Tris-soluble Aβ was 19-fold elevated in the AN-1792 subjects relative to the NI-AD [5].

The ostensible preferential depletion in Aβ_42_ by bapineuzumab immunotherapy may reflect the antibody avidity for a specific conformational domain of the Aβ_42_ peptide. Molecular modeling of Aβ_42_ suggests an ionic bond between the N-terminal amino group of 1Asp and the C-terminal carboxyl group of Aβ_42_Ala [24] at the surface of dimeric Aβ_42_. This intramolecular conformation may have a higher affinity for the antigen-antibody interaction. Removal of dimeric Aβ from the filamentous structures is in all likelihood the way by which these structures are disassembled from AP by antibodies, since the intermolecular bonds within dimers are thermodynamically a billion times more stable than those within free metastable monomers in a misfolded state [25]–[27]. Furthermore, bapineuzumab immunotherapy may be limited in its effects because its specific epitope target is partially or totally absent in a high proportion of deposited Aβ peptides due to the partial N-terminal degradation of these molecules [28], [29] and to the post-translational modifications such as Asp-isomerization [28] and pyroglutamyl-cyclization [30], which enhance polymerization and dimer resistance to enzymatic degradation [31]. In contrast, the apparently more completely disaggregated ‘collapsed’ plaque skeletons observed in AN-1792-immunized individuals [9] could be a consequence of the polyclonal nature of this vaccine which may have efficiently eliminated Aβ_42_ as well as a wide range of other species including N-terminally-degraded Aβ peptides. If it is true that a cascade of deleterious events leading to dementia follows amyloid deposition, it suggests protective elimination of both Aβ_40_ and Aβ_42_ must be preemptive and thorough.

It is important to bear in mind that although bapineuzumab immunotherapy impacted Aβ_42_ levels, it does not apparently suppress the primary impetus for amyloidogenic AβPP processing or deposition. The loss of Aβ_42_ and the noticeable elevation of Aβ_40_ suggest a possible compensatory mechanism in which one form of Aβ is substituted for the other. For example, compensatory deposition of Aβ_40_ may explain the lack of remarkable differences between AP densities in Bapi-AD and NI-AD individuals despite Aβ_42_ depletion. The apparent compensatory production and the observed high levels of vascular amyloid in Aβ-immunized individuals lends support to the hypothesis postulating that one of the functions of Aβ peptides is to generate a protective hemostatic-like patch/scab to seal leaky blood vessels at the abluminal side of the brain microvessels (reviewed in [32]–[35]). Bapineuzumab immunotherapy induces brain effusive vasogenic edema defined as Amyloid Related Imaging Abnormalities (ARIA-E) and microhemorrhages (ARIA-H) in AD individuals [36]. Flair-MRI scans showed that 36 (17%) out of 210 bapineuzumab-treated AD patients developed vasogenic edema and that 17 (47%) of these patients developed microhemorrhages while only seven (4%) patients out of the remaining 177 without ARIA-E developed microhemorrhages [37]. Transient vasogenic edema has been observed in other studies of Bapi-AD subjects [12], [38], [39]. A study of 2762 AD patients at baseline in immunotherapy clinical trials found AD-related vasogenic edema to be rare, occurring in only 2 cases [40]. The generation of microhemorrhages in immunized AβPP Tg mice is a well documented observation [41]–[46]. It is possible that blood-brain barrier (BBB) breaches could have been caused by bapineuzumab or autoantibodies generated by the high levels of Aβ in addition to a vascular inflammatory reaction. Taken together, these observations imply that partial removal of Aβ_42_ from the vascular walls by bapineuzumab immunotherapy leads to a cycle of increased production of Aβ_40_ in an attempt to maintain the integrity of the BBB in the face of immunotherapy-induced alterations.

Interestingly, in the Dominantly Inherited Alzheimer’s Network (DIAN) study, 6% of young asymptomatic FAD mutation carriers showed brain microhemorrhages, and 25% of mildly symptomatic carriers also had these lesions of which 16% had 1–4 and 9% had >5 microhemorrhages [47]. In addition, pure PS Tg mice, without AβPP mutations and hence without AP, demonstrated widespread ultrastructural microvascular pathology [48]. Older AβPP/PS1 Tg mice (19–23 months of age), also developed spontaneous ARIA-E and ARIA-H in areas adjacent to vascular amyloid deposition in the absence of immunotherapy [49], [50].

As the brain ages, inevitable microvascular decline and accumulating damage decrease BBB integrity [51]. Chronic diseases such as hypertension, hypotension, diabetes, atherosclerosis, arteriosclerosis [52], [53] as well as traumatic brain injury [54], [55] have a deleterious effect on brain microcirculation [56]–[59]. Partial ligation of the thoracic aorta in pigs produces both hypertension and a substantial increase in Aβ_40,_ Aβ_42_ and p^412^-tau in the brains of these animals [60], suggesting general perfusion failure synergizes the critical pathological cascades associated with AD. The physical attributes of amyloid fibrils such as their chemical stability, insolubility, amphipathic structure, cement-like properties (reviewed in [32]), metal and heme binding capacity [24], [61]–[64] and their ability to interact with the extracellular matrix to generate resilient meshes, similar to those of the coagulation cascade, make amyloid an ideal brain microvascular repair/protective substance [32], [33], [65]. Moreover, Aβ binds and sequesters plasma molecules that under normal circumstances would be excluded from the brain [66]–[71]. The Aβ peptides also interact with key coagulation molecules including thrombin, fibrinogen and plasminogen [72]–[78] and in this context the AβPP_770_ and AβPP_751_ isoforms containing the Kunitz protease inhibitor domain play an important regulatory role in the coagulation cascade [79], [80]. Moreover, in humans, the complex amyloid deposits appear to be protected against proteolytic degradation by α1-antichymotrypsin [81], [82]. The continuous and excessive accretion of amyloid around the brain microvessels may terminate by constricting the lumen, creating blind capillary stumps and impaired perfusion [32]. It can be postulated that the brain of a patient with mild and moderate AD has attained or is approaching an adaptive equilibrium in which amyloid production has nearly reached a plateau [83]. Under these circumstances, the removal of Aβ_42_ may trigger a compensatory overproduction of Aβ_40_.

Anti-amyloid immunotherapy, in spite of a reduction of AP in some areas of the brain [5]–[9], [84] and/or dramatic alterations in amyloid composition, has consistently failed to yield commensurate changes in the clinical course of dementia [7], [10], [11]. Although clinical trials of ponezumab, bapineuzumab and solanezumab for mild and moderate AD were halted or did not achieve their primary end points [11], [85], [86], other clinical trials using a variety of immune approaches are still being evaluated. The observation that anti-Aβ immunotherapies produce substantial increases in soluble Aβ peptides provides a possible explanation for the weak impact of these interventions against dementia. Soluble/oligomeric Aβ molecules have been isolated from AD brains [87] and these peptides are clearly neurotoxic [88], [89]. In addition, these species have been largely incriminated as playing a central role in the pathogenesis and pathophysiological evolution of AD and, to a certain extent, displaced the insoluble plaque and vascular Aβ as the primary targets for therapeutic intervention. If these tenets are correct, and in view of the present evidence, it would be expected that in immunized AD patients, an increased pool of soluble oligomeric Aβ should aggravate the natural course of the disease. Larger studies enabling more precise evaluation of the role played by soluble/oligomeric Aβ in immunotherapy recipients are awaited.

The Alzheimer’s Prevention (Preclinical) Initiative (API) and the DIAN studies will involve the proactive administration of anti-Aβ immunotherapy treatments to subjects harboring PS mutations years prior to the estimated time of cognitive impairment appearance. Clinical trials have confirmed that anti-amyloid immunotherapy produces a range of considerable positive and negative effects on critical biomarkers of sporadic AD [5]–[9], [12], [14], [39], [84], [90]–[95]. However, whether strategically timed pre-symptomatic treatments will produce analogous responses and/or preserve cognitive function in PS mutation carriers is uncertain. Biochemical studies of PS mutations have led to the widely held hypothesis that enriched production of Aβ_42_ underlies dementia [96], [97], although rigorous quantitation of Aβ peptides in humans with FAD has revealed that this rule is clearly not universal [98], [99]. Whether or not crenezumab, solanezumab or gantenerumab treatments favor Aβ_40_ production and accumulation in FAD-PS mutation carriers, API and DIAN will offer an ideal opportunity to directly test the hypothesis that excess Aβ_42_ production alone specifically initiates dementia emergence. However, several lines of evidence, including the clinically disappointing, but highly informative, outcomes of immunotherapy trials suggest that dementia progression has an unappreciated biochemical complexity. Our study and previous investigations of the molecular aftermath of immunotherapy [5], [9], [14] suggest that Aβ_40_ may not be entirely benign, as elevated quantities of this species are strongly associated with increased vascular amyloidosis and continued decline into dementia. Again, the API and DIAN will provide an ideal prospect to unequivocally examine the potential association of these specific biomarkers with dementia onset and progression as well as a test for the validity of the amyloid cascade hypothesis.

In summary, our study and previous investigations confirm that Aβ immunotherapy profoundly impacts the prime AP and Aβ42 targets. In some cases, immunotherapy resulted in the physical disruption of AP. In the case of our study, although AP appeared unaltered physically, the amyloid profile in treated subjects was radically perturbed with levels of Aβ_42_ substantially decreased and Aβ_40_ levels increased. Despite these responses, immunotherapy did not produce any substantial impact on dementia evolution. The precise mechanism(s) underlying this equilibrium shift is unknown, but the net effect of immunotherapy may be a marked preferential accumulation of the shorter and more soluble Aβ_40_ species. Coupled with the recognized complexity of AD biochemistry and neuropathology, these observations suggest that amyloid plaque deposits either cannot be the sole instigator of cognitive breakdown or their precise molecular constitution is of little overall consequence to dementia appearance. If this is the case, it suggests that despite remarkable impacts on AD biomarkers, the consistent lack of success of immunotherapy against dementia reflects the general failure to understand the function of amyloid deposition and its dynamics comprehensively.
